# Double-tube burr hole irrigation in the treatment of subdural empyema following chronic subdural hematoma surgery: A case report

**DOI:** 10.1016/j.ijscr.2024.109240

**Published:** 2024-01-10

**Authors:** Manato Sakamoto, Shigeomi Yokoya, Midori Ichihashi, Kengo Kishida, Hideki Oka

**Affiliations:** Department of Neurosurgery, Saiseikai Shiga Hospital, Imperial Gift Foundation Inc., Ritto, Shiga, Japan

**Keywords:** Subdural empyema, Burr hole irrigation, Double-tube

## Abstract

**Introduction and importance:**

Subdural empyema (SE) following chronic subdural hematoma (CSDH) surgery is an uncommon but serious complication. The best treatment approach, typically a choice between craniotomy and burr hole surgery, is still debated. This case report introduces an innovative method using burr hole surgery with double-tube irrigation, a potentially effective alternative to the more invasive craniotomy.

**Case presentation:**

An 81-year-old male, 48 days post-CSDH surgery, developed SE with Methicillin-resistant *Staphylococcus aureus* infection. The initial treatment with burr hole drainage was complicated by recurrence, leading to a second procedure with double tubes inserted anteriorly and posteriorly for continuous irrigation therapy. The patient was treated with systemic antibiotics and vancomycin irrigation, resulting in successful resolution without further recurrence.

**Clinical discussion:**

While burr hole surgery is often deemed less effective than craniotomy for SE, this case demonstrates the potential efficacy of double-tube irrigation via burr hole surgery. This method could be especially beneficial when craniotomy poses significant risks. Continuous irrigation could help in managing intracranial pressure, making the intervention safer. However, further research is needed to refine this technique and establish clear treatment guidelines.

**Conclusion:**

Burr hole surgery with double-tube irrigation emerges as a promising treatment option for SE, especially when craniotomy is not feasible. This approach's success in this case encourages further exploration and study to validate its wider application in similar clinical scenarios.

## Highlights

• **Novel Double-Tube Burr Hole Irrigation** - New method for Subdural Empyema treatment.
• **Effective Against Resistant Infections** - Successful in Methicillin-resistant *Staphylococcus aureus* case.
• **Reduces Complications** - Limits risks associated with traditional craniotomy.
• **Continuous Drainage Strategy** - Improves safety and efficacy in SE management.
• **Case Study Insights** - Offers practical implications for patient care.

## 1 Introduction

Subdural empyema (SE) that develops after chronic subdural hematoma (CSDH) is rare but constitutes a significant complication with the potential for severe outcomes. In previous studies, SE has shown a higher recurrence rate when treated with burr hole surgery, suggesting that craniotomy drainage is more effective ([1], [2], [3]). However, craniotomy necessitates general anesthesia and creates a larger surgical wound, increasing the patient's burden (4). The most effective treatment is yet to be universally agreed upon. We report a case where drainage and antibiotics irrigation through burr hole surgery was performed for a recurrence SE post-CSDH surgery, utilizing two drains oriented anteriorly and posteriorly. We declare that the work has been reported in line with the SCARE 2023 criteria (5).

## 2 Case description

**Fig. 1:**
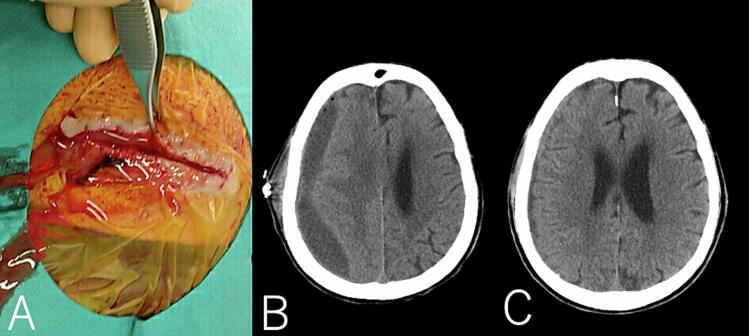
A: Pus was oozing from the wound. B: Head Computed Tomography (CT) reveal a recurrent abscess in the right epidural space. C: No recurrence was observed in the CT scan at the time of discharge.

**Fig. 2:**
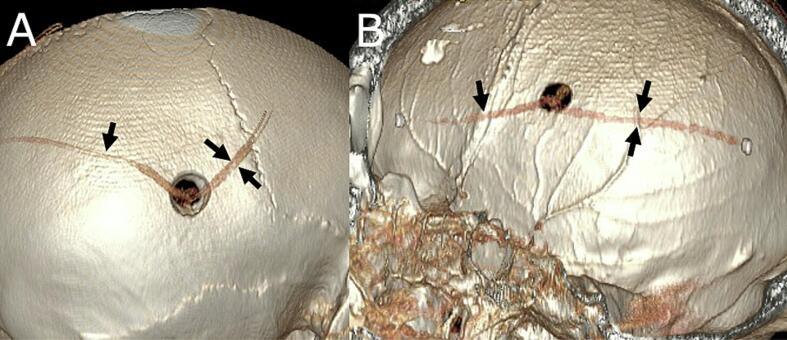
A, B: Reconstructed postoperative cranial CT images. Two drains are inserted anteriorly and posteriorly into the subdural space from a burr hole in the left parietal region. One drain (indicated by the arrow) is used for medication infusion, while the other (indicated by the paired arrow) discharges effluent.

An 81-year-old male, the patient, presenting with incomplete paralysis of the upper and lower left limbs, visited our hospital emergency room. A head computed tomography (CT) scan revealed a right chronic subdural hematoma accompanied by a mass effect. An emergency burr hole surgery was performed on the same day, and the patient was discharged to home on the second day post-operation. Forty-eight days after the initial surgery, the patient revisited our emergency outpatient clinic, presenting with pus discharge from the surgical wound. Neurologically, no abnormalities were detected, and blood tests also showed no anomalies. However, a head CT scan revealed fluid accumulation in the subdural space. Burr hole surgery was performed on the same day under local anesthesia. Postoperatively, the patient was started on systemic vancomycin 3 g/day and cefepime 6 g/day. The drain was removed the next day. Culture of the pus obtained intraoperatively identified *Methicillin-resistant Staphylococcus aureus*, leading to a switch to exclusive vancomycin 3 g/day therapy. Seven days post the empyema drainage surgery, pus again oozed from the surgical site (Fig. 1A), and imaging demonstrated a tendency for reaccumulation in the subdural space, necessitating another drainage procedure (Fig. 1B). This time, two drains were placed anteriorly and posteriorly through a single burr hole. From the next day, the anteriorly placed drain was infused with saline and 20 mg of vancomycin, with effluent being collected from the posterior drain, initiating continuous irrigation therapy (Fig. 2). Over time, the discharge of pus from the drain reduced, allowing for its removal two weeks postoperatively. Subsequent imaging showed no recurrence for 3 month follow-up(Fig. 1C).

## 3 Discussion

Simple burr hole irrigation is often considered insufficient for SE surgery, craniotomy, which involves exposing and removing the entire abscess, is typically preferred to prevent recurrence. However, this approach often necessitates bone flap removal, raising concerns about cranial defects ([1], [2], [3]). Post-empyema resolution, cranioplasty might be required, entailing additional surgery and risk of secondary infection. Our study introduces a groundbreaking approach in the treatment of SE, leveraging double-tube burr hole irrigation. This method contrasts sharply with traditional techniques and offers distinct advantages, potentially circumventing issues associated with craniotomy.

Our case suggests that while single-tube, short-duration drainage might be insufficient, double-tube burr hole irrigation emerges as a viable therapeutic option for empyema. Similar strategies have been recognized in fields like cervical surgery, where the insertion of two drains for irrigation in infection treatment is acknowledged (6,7). Recent reports also highlight the augmentation of burr hole surgery with neuroendoscopy (8), suggesting that combining this technique with our double-tube irrigation could further enhance treatment efficacy.

While addressing the immediate risks of drain malfunction is crucial for patient safety, another aspect of concern is the optimal duration for drain placement, a topic still under debate. If the draining drain isn't working, there's a potential risk that irrigation from the infusion drain could endanger the patient. Hence, it's essential to check for blockages during irrigation. However, if discharge is achievable even with a single drain, the method is considered relatively safe in terms of not leading to excessive intracranial pressure elevation.

In our case, the drain was left in place for two weeks postoperatively. Decisions regarding drain removal were based on culture results of the drain effluent and imaging studies. Extended drain placement can complicate management and hinder patient mobilization, significantly impacting activities of daily living, especially in older patients. While our approach shows promise, it's important to note the limitations of this study, including its single-case focus and the absence of a control group. Future research should aim to validate these findings in a broader clinical context.

Furthermore, the irrigation therapy used saline and an antibiotic (vancomycin) to which the identified bacteria were sensitive. The choice between using saline alone or an antibiotic-mixed saline remains unsettled, and the quantity and concentration of antibiotics introduced intracranially are also of concern, with no established guidelines (9).

## 4 Conclusion

For SE, burr hole surgery employing double-tube irrigation might be considered a viable therapeutic option.

## Consent

Written informed consent was obtained from the patient for publication of this case report and accompanying images. A copy of the written consent is available for review by the Editor-in-Chief of this journal on request.

## Ethical approval

Written informed consent was obtained from the patient for publication of this case report and accompanying images. Ethical approval for this study (Ethical Committee N° 597) was provided by the Ethical Committee of Saiseikai Shiga Hospital, Ritto, Shiga, Japan on 14 November 2023.

## Funding

Nil.

## Author contribution

Study concept or design: Manato Sakamoto, Shigeomi Yokoya, Midori Ichihashi, Kengo Kishida, Hideki Oka.

Data collection: Manato Sakamoto.

Data analysis or interpretation: Manato Sakamoto.

Writing the paper: Manato Sakamoto, Shigeomi Yokoya.

## Guarantor

Manato Sakamoto.

## Declaration of competing interest

There is no conflict of interest.
